# Cloning and analysis of *PRNP* gene of *Vulpes corsac* in Qinghai plateau, China

**DOI:** 10.1080/19336896.2019.1704496

**Published:** 2019-12-27

**Authors:** Xue-Hua Yang, Kang Xiao, Yuezhang Wu, Liping Gao, Dongdong Chen, Xiao-Ping Dong, Qi Shi

**Affiliations:** a State Key Laboratory for Infectious Disease Prevention and Control, Collaborative Innovation Center for Diagnosis and Treatment of Infectious Diseases (Zhejiang University), National Institute for Viral Disease Control and Prevention, Chinese Center for Disease Control and Prevention, Beijing, People’s Republic of China; b Center for Global Public Health, Chinese Center for Disease Control and Prevention, Beijing, China; c Center for Biosafety Mega-Science, Chinese Academy of Sciences, Wuhan, People’s Republic of China

**Keywords:** *PRNP*, *Vulpes corsac*, prion disease, Qinghai plateau

## Abstract

*PRNP* gene encodes PrP protein, which is conservative among different species and associates with the susceptibility of prion disease. In this report, we cloned and sequenced the full-length *PRNP* gene of *Vulpes corsac* in Qinghai plateau, China. The amino acid sequence of *Vulpes corsac* PrP showed 100% homology with those of the other three species of foxes. The taxa relationship of *Vulpes corsac* PrP with other species of animals, including human, canine, bovine, cervus, capra, ovis, camelus, felis, Mustela, mouse and hamster were also analysed.

## Introduction

Prion diseases, or transmissible spongiform encephalopathies (TSE), are a group of transmissible, fatal neurodegenerative diseases affecting a wide variety of mammals including humans [1,2]. The conversion of the normal and non-infectious cellular form of the host prion protein (PrP^C^) into the abnormal and pathogenic form (PrP^Sc^) is critical for prion disease [3]. Prion protein (PrP) is encoded by a host gene, namely *PRNP*, usually contains one open reading frame (ORF) [4]. Despite that the sequence of *PRNP* is fairly conservative among different species of animals, the full extent of *PRNP* allele is closely associated with the susceptibility of the infections of different prion strains both naturally and experimentally [5–8].

*Vulpes corsac* belongs to fox species, which mainly inhabits steppe, desert and semi-desert areas and distributes in Central Asia, ranging into Mongolia and northern China [9]. This kind of animal has three subspecies including *Vulpes corsac corsac, Vulpes corsac kalmykorum* and *Vulpes corsac turcmenicus* [10]. In this report, we have described the full-length of *PRNP* gene of *Vulpes corsac* that was captured in Qinghai plateau, China.

## The sequence of *Vulpes corsac* PrP

The genomic DNA from the liver tissue of a natural death Corsac fox collected in Qinghai province was extracted using QIAamp DNA Mini Kit. The *PRNP* sequence was amplified by PCR technique with the designed primers (upstream primer: 5`-ATGGTGAAAAGCCACATAG-3`; downstream primer: 5`-TCATCCCACTATCAAGAGA-3) based on the *PRNP* sequences of *Vulpes vulpes* (EF571898), *Vulpes velox* (EU341513) and *Vulpes lagopus* (EU365392) in NCBI website. The reaction conditions were 94°C for 1 min, 52°C for 30 s, 72°C for 40 s, totally 35 cycles. After purification, the PCR product was inserted into a clone vector pMD19 and sequenced with the primer designed according to the sequence of the cloning vector.

**Figure 1.**
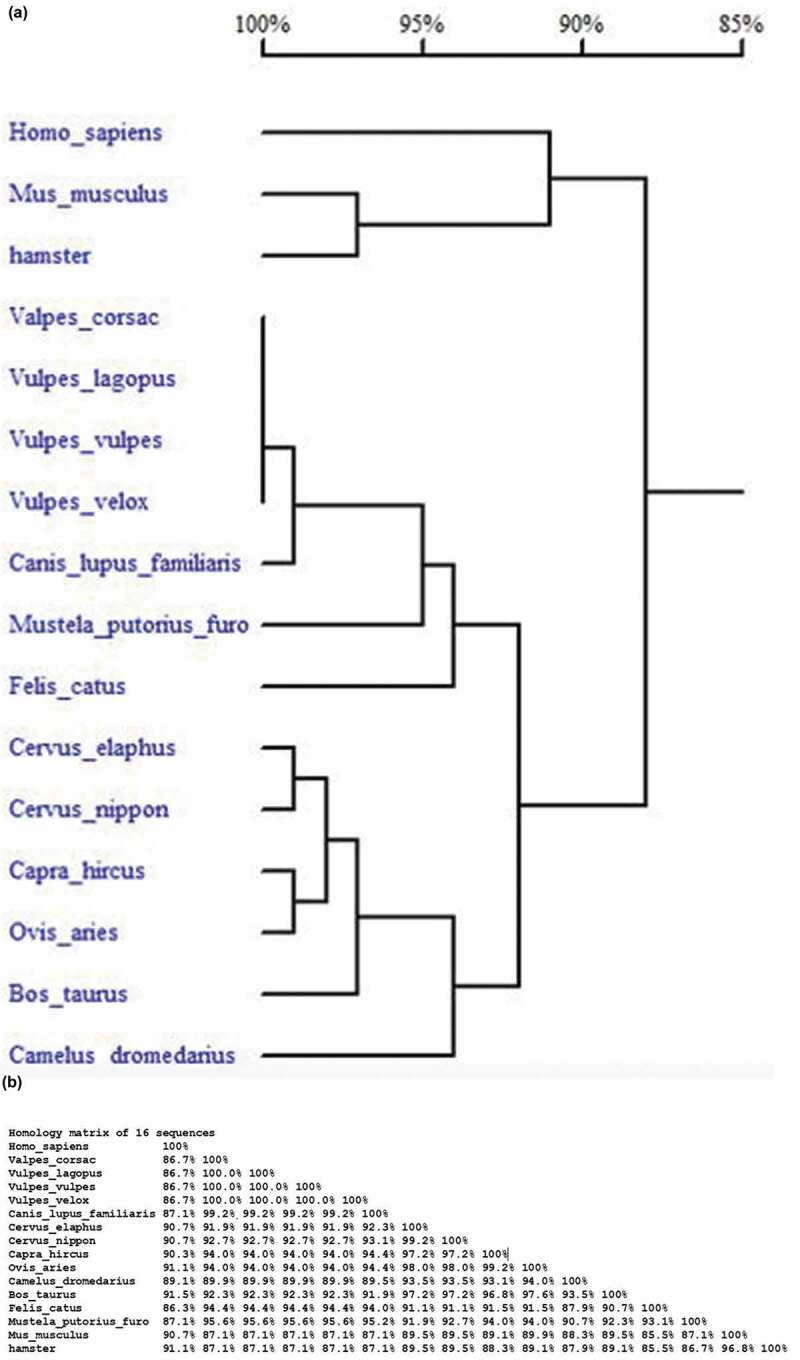
Homology analysis of amino acid sequences of PrP proteins of human and various species of animals. (a). Phylogenetic tree. (b). Homology matrix.

According to the sequencing results, the *PRNP* sequence of the tested *Vulpes corsac* was 774 bp long, which may encode 257 amino acids (submitted to NCBI, MN381732). Compared with the published data of foxes, including red fox, Swift fox and Arctic fox, the amino acid sequence of *Vulpes corsac* PrP was 100% homology. The homology of *Vulpes corsac* PrP with human and other species of animals was also illustrated in the phylogeny tree (Figure 1(a)). It revealed an extremely close homology with canine PrP (NP_001013441), while apparently remote relationship with the PrP of human (NP_898902). Meanwhile, the PrP sequences of *Vulpes corsac*, as well as other foxes and dog, showed also remote relationship with that of the animal species with naturally occurred prion diseases, such as cattle (ABE02802), ovis (NP_001009481, NP_001301176), cervus (QAU19537, AAT72295), cat (ACA50727), ferret (XP_012901521) and camel (AUM59985), as well as the animals with experimental prion diseases, such as mouse (NP_001265185) and hamster (AAA37013).

The homology matrix of human, *Vulpes corsac* and other species of mammalia is illustrated in Figure 1(b). Beside of the canine PrP showing 99.2% homology, PrP of *Vulpes corsac* revealed 95.6% homology with that of *Mustela putorius*, 94.4% with feline, 94.0% with ovis, 92.7% with *Cervus nippon*, 92.3% with *Bos taurus*, 91.9% with *Cervus elaphus*, 89.9% with camel, 87.1% with mouse and hamster, and 86.7 with human.

**Figure 2.**
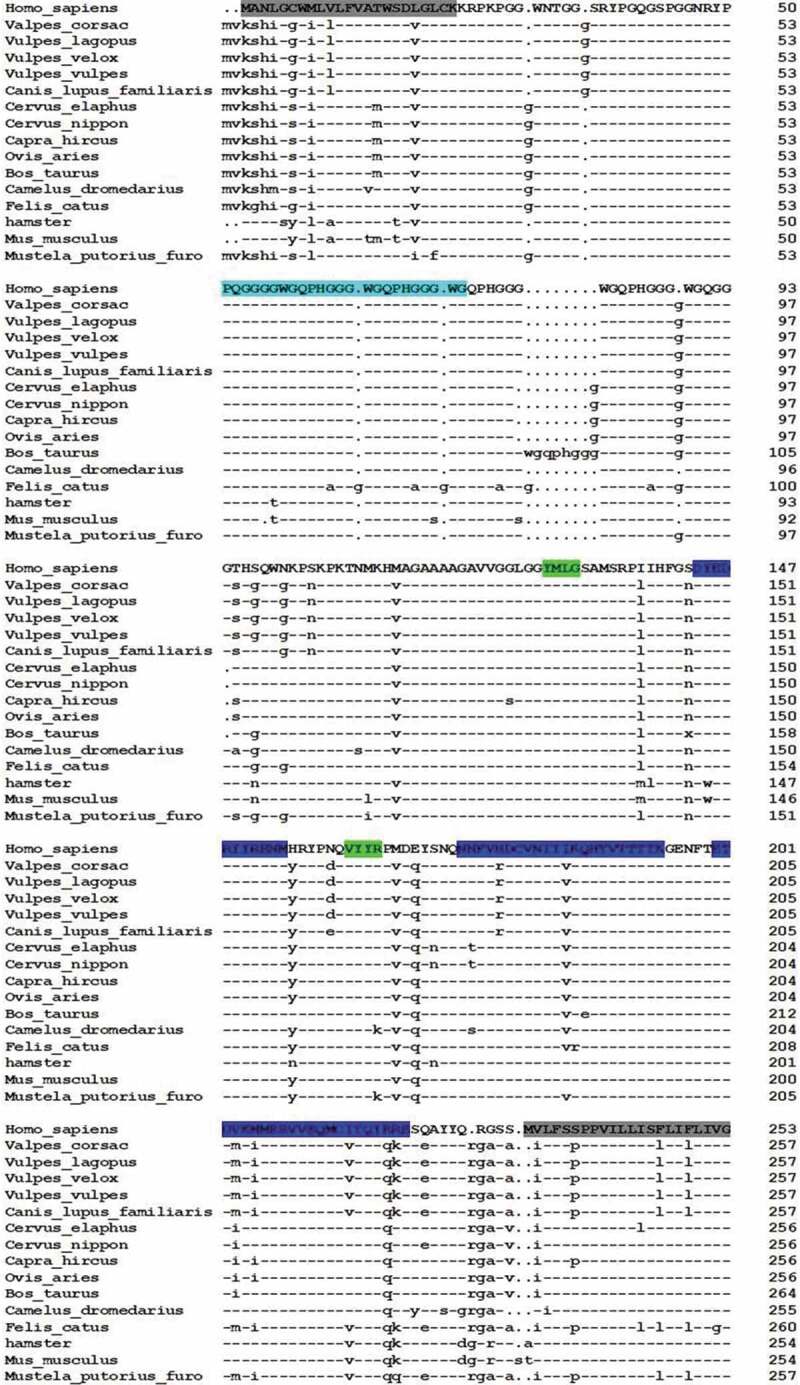
Comparison of the variations of amino acids of PrP proteins of human and various animals. The various functional and secondary structural regions within PrP sequences are indicated with colours. The amino acid numbers are shown on the right.

The exact differences of PrP sequences among the different species are shown in Figure 2. Similar to that of the fox, canine PrP consisted of 257 amino acids. Only two amino acids were different between fox and dog, which located at the position of aa 101 from Gly (fox) to Ser (dog) and at the position of aa 163 from Asp (fox) to Glu (dog), indicating a high homology of PrP within the canine family. Human PrP consists of 253 amino acids. There were 36 amino acid differences in PrP peptides between the *Vulpes corsac* and human. The regions with more discrepancies in amino acid between fox and human PrPs were N-terminal signal sequence (39.2%) and C-terminal GPI anchor (22.7%). Amino acid variations were also identified within the regions of α3 (22.7%) and α2 (9.1%), but not in the regions of α1, β1 and β2. Further comparisons of the sequence of *Vulpes corsac* PrP with those of bovine and cervus PrPs also revealed the similar variation patterns. Besides of differences in the regions of the signal peptide and GPI anchor, variations were also found in the regions of α3 (18.1%) and α2 (9.1%), but not in those of α1, β1 and β2.

## Discussion

In this report, we have for the first time described the *PRNP* sequence of *Vulpes corsac* in Qing-Tobent plateau. *Vulpes corsac* belongs to canine family, fox subfamily, fox genus. Generally, the animals of canine species are not sensitive to prion infection. So far, there is no naturally occurred canine TSE reported [11,12], including the animals of Vulpes. Among the factors that may contribute to the susceptibility or resistance to the prion strains, the host PrP sequence is believed as the most essential one. The high identity of *PRNP* sequence between *Vulpes corsac* and dog indicates that *Vulpes corsac* may also be insensitive to prion infection.

*Vulpes corsac* distributes widely in the different geographies of Central Asia and the western region of China, usually taking rodents and birds as the main source of food, such as yellow mouse, striped hamster and Brandt’s vole. Meanwhile, many other carnivores may prey *Vulpes corsac*, such as caracal, jackal, etc. Qinghai-Tibet plateau is an important pastoral area in China. There are thousands of livestock, e.g., yaks, sheeps and goats, as well as wild herbivore, e.g., Tibetan antelope, kiang, wild yak, mongolian gazelle, etc. As a middle link of food cycle in a special geography, understating the sequence of *Vulpes corsac* PrP may help for evaluation its potential in the circulation of prions, e.g., scrapie, in a special region.

## References

[1] Colby DW, Prusiner SB. Prions[J]. Cold Spring Harb Perspect Biol. 2011;3(1):13363–13383. 10.1101/cshperspect.a006833 PMC3003464 21421910

[2] Prusiner SB. Nobel lecture: prions[J]. Proc Natl Acad Sci U S A. 1998;95(23):13363–13383. 9811807 10.1073/pnas.95.23.13363 PMC33918

[3] Westergard L, Christensen HM, Harris DA. The cellular prion protein (PrP(C)): its physiological function and role in disease[J]. Biochim Biophys Acta. 2007;1772(6):629–644. 17451912 10.1016/j.bbadis.2007.02.011 PMC1986710

[4] Prusiner SB. Molecular biology of prion diseases[J]. Science. 1991;252(5012):1515–1522. 1675487 10.1126/science.1675487

[5] Hadlow WJ, Race RE, Kennedy RC Experimental infection of sheep and goats with transmissible mink encephalopathy virus.[J]. Can J Vet Res Revue canadienne de recherche vétérinaire. 1987;51(1):135. 2952237 PMC1255287

[6] Hanson RP, Eckroade RJ, Marsh RF, et al. Susceptibility of mink to sheep scrapie.[J]. Science. 1971;172(3985):859–861. 4102123 10.1126/science.172.3985.859

[7] Robinson MM, Hadlow WJ, Huff TP, et al. Experimental infection of mink with bovine spongiform encephalopathy.[J]. J Gen Virol. 1994;75(Pt 9):2151. 8077914 10.1099/0022-1317-75-9-2151

[8] Robinson MM, Hadlow WJ, Knowles DP, et al. Experimental infection of cattle with the agents of transmissible mink encephalopathy and scrapie[J]. J Comp Pathol. 1995;113(3):241–251. 8592050 10.1016/s0021-9975(05)80039-8

[9] Murdoch JD 2014. Vulpes corsac. The IUCN red list of threatened species 2014: e.T23051A59049446. [EB/OL]. [cited 2019 77]. Available from: https://www.iucnredlist.org/en

[10] Search|BioLib.cz[EB/OL]. [cited 2019 77]; Available from: https://www.biolib.cz/en/formsearch/?action=execute&searcharea=1&string=Vulpes+corsac

[11] Sanchez-Garcia J, Fernandez-Funez P. D159 and S167 are protective residues in the prion protein from dog and horse, two prion-resistant animals[J]. Neurobiol Dis. 2018;119:1–12. 30010001 10.1016/j.nbd.2018.07.011 PMC6139044

[12] Won SY, Kim YC, Kim K, et al. The first report of polymorphisms and genetic features of the prion-like protein gene (PRND) in a prion disease-resistant animal, dog[J]. Int J Mol Sci. 2019;20(6):1404. 10.3390/ijms20061404 PMC6470729 30897750

